# Supplementary material for: Phenotypical Flexibility of the EGFRvIII-Positive Glioblastoma Cell Line and the Multidirectional Influence of TGFβ and EGF on These Cells—EGFRvIII Appears as a Weak Oncogene
Source: Int J Mol Sci. 2022 Oct 12;23(20):12129. doi: 10.3390/ijms232012129 (PMC9603514; doi:10.3390/ijms232012129)
Supplement: Supplementary file 1 [file ijms-23-12129-s001.zip › ijms-1899173-supplementary.pdf]

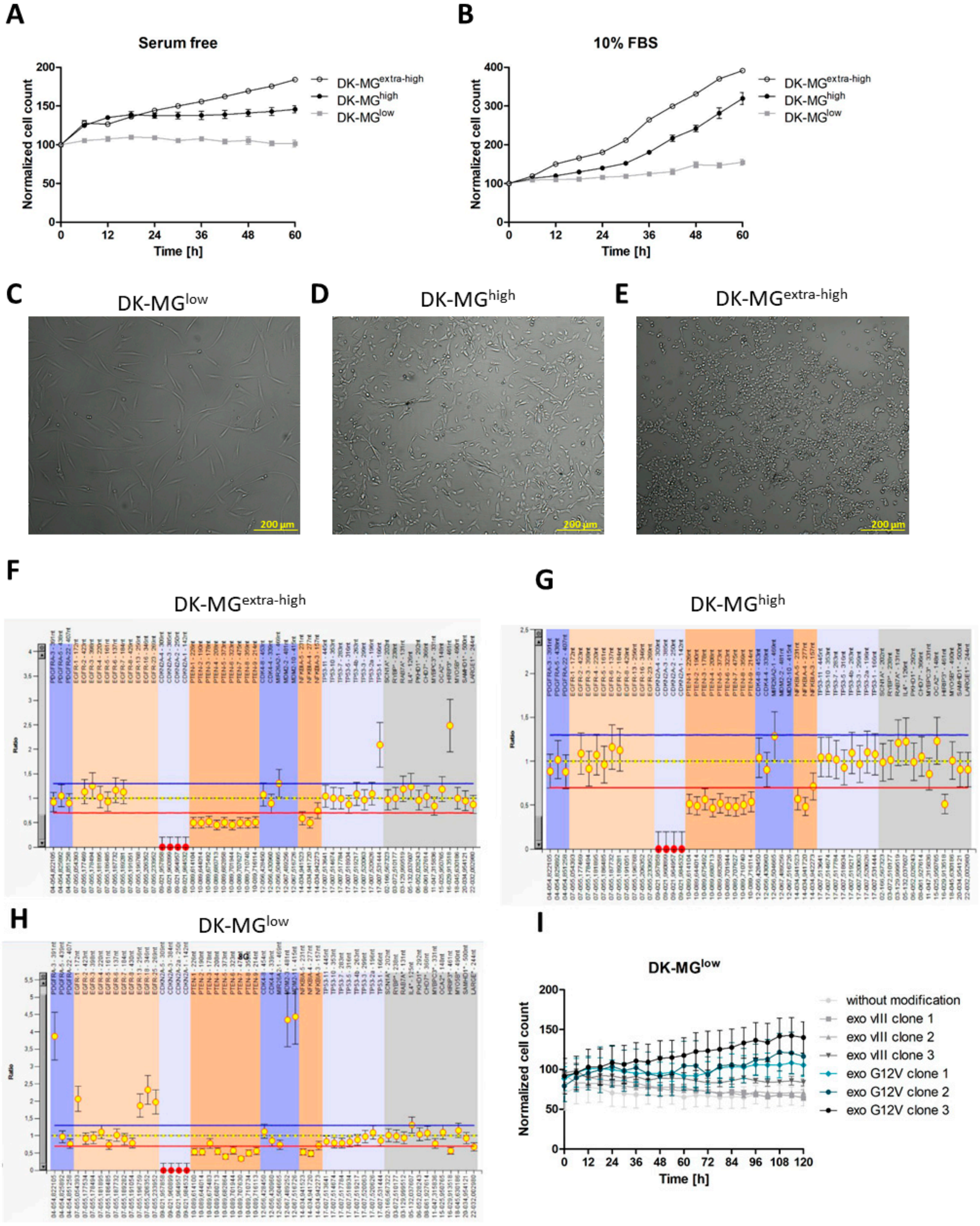

Figure S1. Comparison of the proliferation, phenotype, and genotype (CNV) of various DK-MG sublines. Proliferation ratio of DK-MG^{low}, DK-MG^{high}, and DK-MG$^{extra\text{-}high}$ both in serum-free conditions (A) and full medium (B). Phenotypic changes between different DK-MG sublines were determined microscopically (C–E). MLPA analysis of DK-MG$^{extra\text{-}high}$ (F), DK-MG^{high} (G), and DK-MG^{low} (H) cells revealed no significant differences between sublines as shown on the graph 1D.

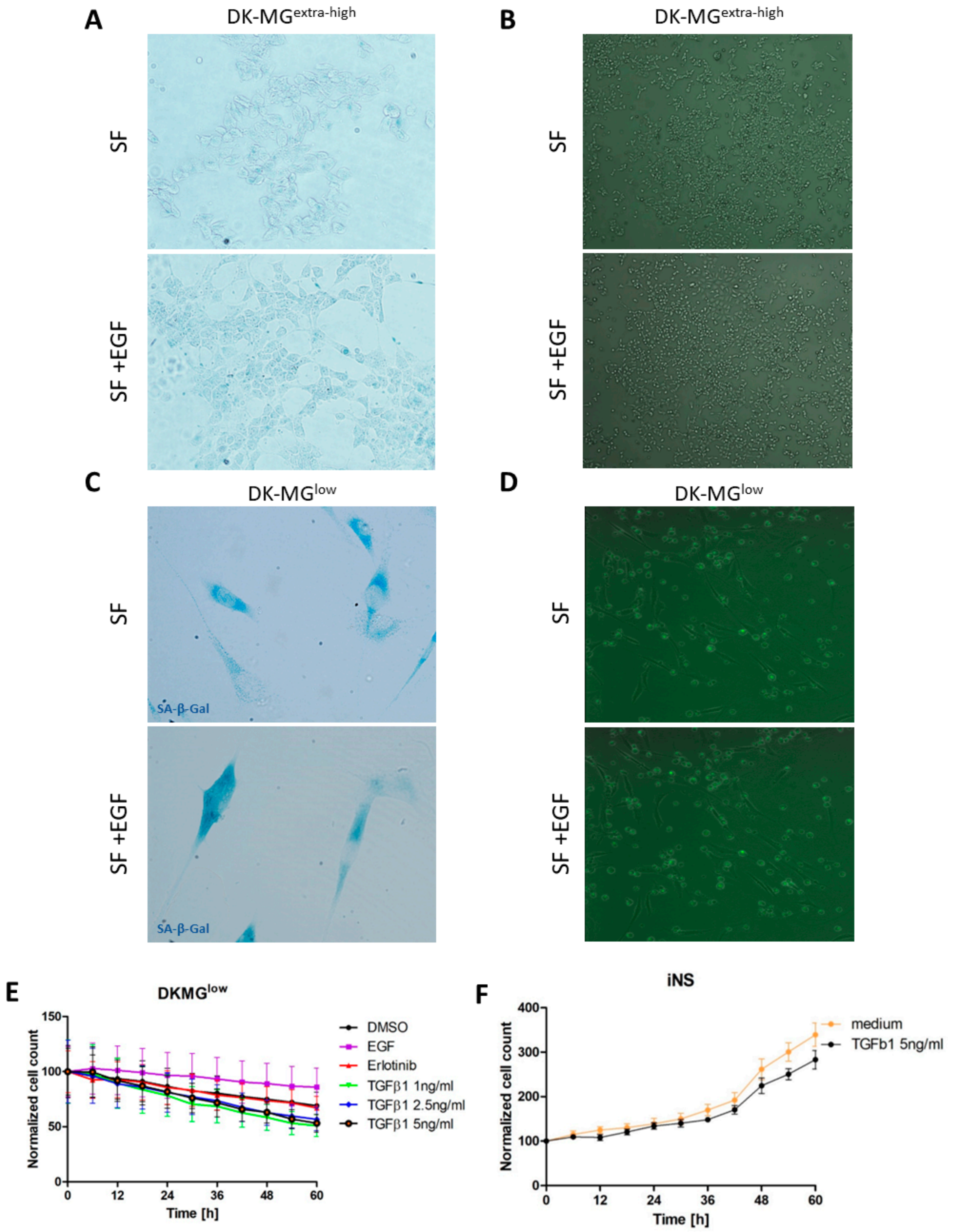

Figure S2. The influence of analyzed growth factors on different cell lines. Impact of epidermal growth factor on the induction of senescence (magnification 400x) (A) and apoptosis (magnification 40x) (B) in DK-MG$^{extra\text{-}high}$ glioblastoma cells after 72 h of treatment with EGF (SF + EGF) or w/o EGF (SF). EGF influence on senescence induction (magnification 600x) (C) and apoptosis in DK-MG^{low} subline after 72 h-incubation with epidermal growth factor (SF + EGF) and in serum-free conditions (SF); magnification 40x (D). The influence of TGFβ1 on normal cells–treatment with cytokine had no any negative effect on the proliferation ratio and viability in DK-MG^{low} subline (E) as well as on the induced neural stem cells (F). SF–serum free medium/serum-free conditions.

Table S1. Antibodies used in immunofluorescence and Western blot analyses.

Antibody	Host	Manufacturer	Dilution
BrdU	Mouse	Sigma-Aldrich (B 8434)	1: 500
EGFRvIII	Rabbit	Absolute Antibody (L8A4)	1:500
EGFRvIII	Mouse	Kerafast (L8A4)	1:300
EGFR	Mouse	Santa Cruz Biotechnology (A-10, sc-373746)	1:500
ERK 1/2	Mouse	Santa Cruz Biotechnology (C-9, sc-514302)	1:500
pERK 1/2	Mouse	Santa Cruz Biotechnology (E-4, sc-7383)	1:500
pEGFR Y1068	Rabbit	Cell Signaling (D7A5)	1:500
Akt	Rabbit	Cell Signaling (#9272)	1:500
pAkt S473	Rabbit	Cell Signaling (D9E)	1:500
Actin	Mouse	Millipore (MAB1501, clone 4)	1:4000
anti-rabbit IgG-HRP	Goat	Santa Cruz Biotechnology (sc-2004)	1:4000
anti-mouse IgG-HRP	Goat	Santa Cruz Biotechnology (sc-2005)	1:4000
anti-mouse Alexa Fluor 594	Donkey	Life Technologies	1:500
anti-rabbit Alexa Fluor 488	Donkey	Life Technologies	1:500
